# Recurrent copy number alterations in young women with breast cancer

**DOI:** 10.18632/oncotarget.24336

**Published:** 2018-01-29

**Authors:** Chen Chi, Leigh C. Murphy, Pingzhao Hu

**Affiliations:** ^1^ Department of Biochemistry and Medical Genetics, University of Manitoba, Winnipeg, Manitoba, Canada; ^2^ The George and Fay Yee Centre for Healthcare Innovation, University of Manitoba, Winnipeg, Manitoba, Canada; ^3^ Research Institute of Oncology and Hematology, Cancer Care Manitoba, Winnipeg, Manitoba, Canada; ^4^ Department of Electrical and Computer Engineering, University of Manitoba, Winnipeg, Manitoba, Canada

**Keywords:** recurrent copy number alterations, breast cancer, young women, risk genes, graph algorithm

## Abstract

Breast cancer diagnosis in young women has emerged as an independent prognostic factor with higher recurrence risk and death than their older counterparts. We aim to find recurrent somatic copy number alteration (CNA) regions identified from breast cancer microarray data and associate the CNA status of the genes harbored in the regions to the survival of young women with breast cancer.

By using the interval graph-based algorithm we developed, and the CNA data consisting of a Discovery set with 130 young women and a Validation set with 125 young women, we identified 38 validated recurrent CNAs containing 39 protein encoding genes. CNA gain regions encompassing genes *CAPN2*, *CDC73* and *ASB13* are the top 3 with the highest occurring frequencies in both the Discovery and Validation dataset, while gene *SGCZ* ranked top for the recurrent CNA loss regions. The mutation status of 9 of the 39 genes shows significant associations with breast cancer specific survival. Interestingly, the expression level of 2 of the 9 genes, *ASB13* and *SGCZ*, shows significant association with survival outcome. Patients with CNA mutations in both of these genes had a worse survival outcome when compared to patients without the gene mutations. The mutated CNA status in gene *ASB13* was associated with a higher gene expression, which predicted patient survival outcome. Together, identification of the CNA events with prognostic significance in young women with breast cancer may be used in genomic-guided treatment.

## INTRODUCTION

Although young women only account for 7% of all breast cancers, it is the most common cancer among young females [1]. Yet, young age at diagnosis of breast cancer has emerged as an independent factor for higher recurrence risk and death in various studies [2–6]. Breast cancer in young women has been described to have more biologically aggressive tumours (basal and HER2-enriched subtypes) than in older counterparts, which has been associated with a poorer prognosis [6]. Several factors influence poor prognosis in the young subgroup, such as higher tumour grade at diagnosis, high tumour proliferation, increased expression of HER-2 (*ERB-B2*) and reduced expression of both estrogen (ER) and progesterone receptor (PR) [7]. These women often struggle with life issues that are either absent or much less severe in older women, such as the possibility of early menopause and effects on fertility. While clinicopathologic differences point to underlying biological differences between breast tumours found in younger versus older women, limited studies have documented age-related changes at the molecular level.

Cancer progression is impelled by the accumulation of somatic genetic mutations, which consists of single nucleotide substitutions, translocations and somatic mutations [8]. Somatic mutations are non-heritable alterations to the human genome that occur spontaneously in somatic cells, which is often due to DNA replication error or chemical/ultraviolet (UV) radiation. Copy number alterations (CNA) are somatic changes in the copy numbers of a DNA sequence that arise during the process of cancer development. They consist of changed chromosome structure in the form of gain or loss in copies of DNA segments, and are prevalent in many types of cancer [9]. Investigating these genomic alterations in breast cancer patients can not only offer valuable insights into breast cancer pathogenesis and discover potential biomarkers, but also provide novel drug targets for better therapeutic treatment options [10]. Several cytogenetic and array-based studies have detected recurrent alterations linked with certain cancer types, and have found CNAs to be a particularly common genetic mutation in cancer [11, 12]. In addition, some of these CNAs have resulted in the discovery of disease causal genes and novel therapeutic targets, and have been strongly associated with clinical phenotypes [13–16]. For example, the use of vemurafenib to inhibit BRAF V600E mutation has shown remarkably improved survival in melanoma patients [17]. In another study, treatment with tyrosine kinase inhibitors for EGFR in lung cancer has also shown great success [18].

Since CNAs often encompass genes, it is suspected that they may greatly influence gene expression within the CNA regions. Indeed, several studies have reported a correlation between CNA and the average global expression levels of genes located within the copy number variable chromosomal regions. For instance, one group has shown that in tumour formation from an immortalized prostate epithelial cell line, 51% of genes with increased expression were mapped to DNA gain regions and 42% of genes with decreased expression were mapped to DNA loss regions [19]. This was further supported by another group working with breast tumour cell lines, noting that DNA copy number influences gene expression across a range of CNAs, with 62% of amplified genes resulting in moderately or highly elevated expression of the genes within the amplified regions [20].

Therefore, investigation of CNAs offers the potential to gain insight into the underlying genetic composition of breast tumours in young women. Mining genome-wide profiles will help find breast cancer genes and pathways with strong potential for prognostic significance as a function of age. Given that approximately 40–50% of young breast cancer patients relapse after 5 years [21], these age-specific signatures could also serve as a treatment decision tool to identify young patients that would gain more benefit from particular adjuvant therapies.

## RESULTS

### Clinical characteristics

The young patients with breast cancer in the Discovery and Validation Data sets retrieved from the Molecular Taxonomy of Breast Cancer International Consortium (METABRIC) [22] have very similar distribution in age, menopausal status, tumour grade, tumour size, ER, PR expression and HER2 expressions (*p* > 0.05) (Table 1). On the other hand, the two sets have statistically significant differences in the tumour stage, with young patients in the Discovery set having a much higher prevalence in stage 0 compared to the Validation set (43.1% vs 0.8%) and PAM50 subtypes (*p* < 0.05). However, an overall pattern of the basal subtype being the most frequent amongst young patients is apparent in both the Discovery and Validation dataset. It must be indicated that there are 50 patients in Validation set without stage information, which may affect the analysis of difference in distribution of stages between the two sets. Since our focus is only on those Discovery set CNA candidates that are validated in the Validation set, the stage difference is unlikely to be driving CNA selection. Furthermore, it is our intention to investigate whether tumours in young women share commonalities in genetic alterations, regardless of stages and subtypes.

**Table 1:** Clinical characteristics table comparing the METABRIC discovery dataset and validation dataset for young patients only

Characteristic	Discovery Young	Validation Young	^†^*P*-value
Age^*^	40 (36, 43)	40 (37, 43)	1
**Menopausal Status**			0.5
*Pre*	127 (97.7%^**^)	125 (100%)	
*Post*	2 (1.5%)	0 (0.0%)	
**Subtype**			<0.001
*Normal*	11 (8.5%)	25 (20%)	
*LumA*	41 (31.5%)	18 (14.4%)	
*LumB*	20 (15.4%)	9 (7.2%)	
*Her2*	16 (12.3%)	21 (16.8%)	
*Basal*	42 (32.3%)	52 (41.6%)	
**Grade**			0.98
*1*	7 (5.4%)	6 (4.8%)	
*2*	37 (28.5%)	34 (27.2%)	
*3*	86 (66.1%)	81 (64.8%)	
**Stage**			<0.001
*0*	56 (43.1%)	1 (0.8%)	
*1*	25 (19.2%)	28 (22.4 %)	
*2*	42 (32.3%)	35 (28.0%)	
*3*	7 (5.4%)	11 (8.8%)	
*4*	0 (0.0%)	0 (0.0%)	
**ER-expr**			0.09
*+*	74 (56.9%)	57 (45.6%)	
*–*	56 (43.1%)	68 (54.4%)	
**PR-expr**			0.78
*+*	55 (42.3%)	56 (44.8%)	
*–*	75 (57.7%)	69 (55.2%)	
**Her2-expr**			0.27
*+*	22 (16.9%)	29 (23.2%)	
*–*	108 (83.1%)	96 (76.8%)	
Tumour Size^*^ (mm)	22 (16,30)	(17,30)	1

^*^For continuous variables (Age, Tumour size), quantiles (50th percentile (25th percentile, 75th percentile)) were presented.

^†^*P*-values were determined by Wilcoxon rank sum test for continuous variables and Fisher’s exact test for categorical variables.

^**^The proportion was obtained by dividing the total number of patients.

### Identification of recurrent CNA regions

**Figure 1:**
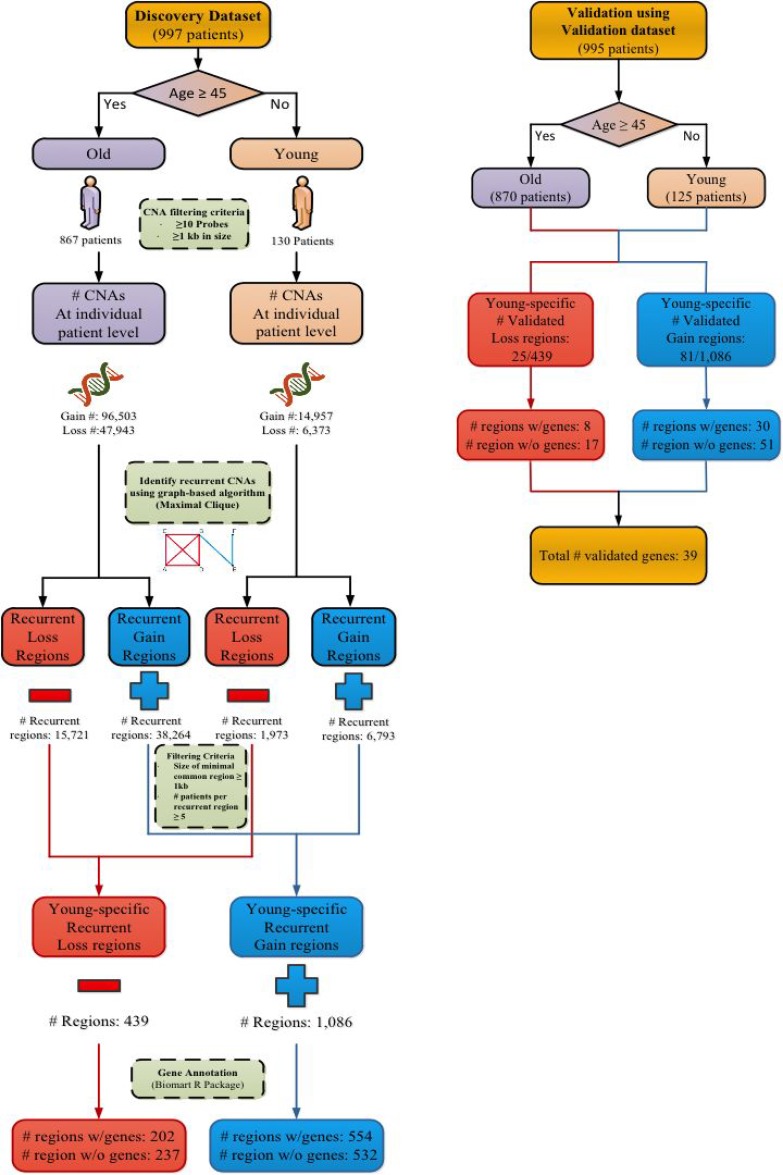
Analysis flowchart for identifying recurrent CNA regions Recurrent CNA regions are identified from young and old patient cohorts in the Discovery set of METABRIC. The identified recurrent CNA regions are then validated in the Validation set of METABRIC.

Figure 1 shows the analysis flowchart to identify age-related recurrent CNA regions using our maximal clique-based recurrent CNA detection algorithm. In the METABRIC Discovery cohort 867 of the total 997 patients are classified into the old age group (≥45 years old) and 130 patients into the young age group. In the Validation cohort 870 of the total 995 patients are classified into the old age group and 125 patients into the young age group. After applying filtering criteria (retaining CNA data that was generated by ≥10 probes and having a CNA size of at least 1 kb), for the old age cohort in the Discovery set, there are 96,503 and 47,943 individual patient level CNA gain and loss regions respectively. For the young age cohort, there are 14,957 and 6,373 individual patient level CNA gain and loss regions, respectively.

Upon filtering for recurrent CNA regions of at least 1 kilobase (kb) in size and having at least 5 patients per recurrent region identified from the recurrent CNA calling algorithm, there are a total of 1,086 recurrent CNA gain regions (554 of the 1,086 gain regions encompassing protein encoding genes) and 439 recurrent CNA loss regions (202 of the 439 loss regions encompassing protein encoding genes). These regions are uniquely found in the young age group and form the young-specific recurrent gain and loss regions in the Discovery set.

Validation testing is then performed using the Validation set, which contains 995 patients. All filtering criteria and algorithm implementations follow the same procedure as the Discovery dataset analysis. For recurrent CNA gain regions, a total of 81 of the 1,086 regions have been validated (found in both the Discovery and Validation datasets), in which 30 regions have encompassed 29 unique protein encoding genes (Table 2). For recurrent CNA loss regions, a total of 25 of the 439 regions have been validated, in which 8 regions encompassed 10 unique protein encoding genes (Table 3). In total, 38 validated recurrent CNA regions with 39 protein encoding genes were identified, along with 51 validated recurrent gain CNA regions (Supplementary Table 1) and 17 validated recurrent loss CNA regions (Supplementary Table 2) that did not encompass any protein encoding genes.

**Table 2:** Validated recurrent gain CNA regions with genes

Chr	Inner Start	Inner End	Inner Size	Outer Start	Outer End	Outer Size	Gene Symbol	Size1	Size2
1	84551640	84565561	13921	84481190	84729446	248256	SAMD13	5	6
1	143607802	143609034	1232	143607067	143609055	1988	PDE4DIP	20	30
1	191374290	191385577	11287	191359529	191405183	45654	CDC73	40	50
1	191385797	191402004	16207	191359529	191405183	45654	CDC73	40	50
1	222004315	222004925	610	221990859	222005526	14667	CAPN2	48	47
3	176423680	176427601	3921	176415397	176428705	13308	NAALADL2	18	22
3	176428607	176428705	98	176415397	176470832	55435	NAALADL2	18	22
5	22246497	22346803	100306	22194503	22414425	219922	CDH12	12	11
6	34625387	34634997	9610	34624907	34656516	31609	SPDEF	9	9
7	134782461	134787038	4577	134782461	134792291	9830	CNOT4	11	13
7	142150844	142154230	3386	142150819	142154515	3696	PRSS1	7	10
8	40695071	40697114	2043	40693570	40699795	6225	ZMAT4	17	16
9	93166194	93261927	95733	93157333	93373420	216087	NFIL3	5	7
10	5737990	5742226	4236	5736767	5744158	7391	ASB13	24	32
10	14598341	14600566	2225	14477106	14629604	152498	FAM107B	19	27
11	4931741	4932834	1093	4931741	4932966	1225	MMP26; OR51A2	7	11
12	180797	191614	10817	180797	195197	14400	SLC6A12	14	24
12	7895693	7897774	2081	7895693	7897774	2081	SLC2A14	13	23
12	7899067	7905082	6015	7899067	7909593	10526	SLC2A14	13	23
13	112354883	112363586	8703	112333434	112363586	30152	C13orf35	10	7
13	113356126	113365589	9463	113345036	113371998	26962	ATP4B	10	9
17	43751830	43753351	1521	43722185	43756717	34532	SKAP1	9	14
17	45136676	45139395	2719	45134175	45142242	8067	SLC35B1	21	18
18	9549925	9575313	25388	9417006	9594232	177226	PPP4R1	5	7
18	43704001	43707399	3398	43703934	43707399	3465	SMAD2	6	6
19	40629439	40647918	18479	40620640	40702499	81859	FFAR2	13	13
19	60890917	60901410	10493	60890917	60904859	13942	EPN1	8	11
20	14741416	14743670	2254	14741416	14743754	2338	MACROD2	8	10
20	41215727	41219453	3726	41202818	41220578	17760	PTPRT	12	18
22	20146692	20170596	23904	20145867	20170766	24899	PI4KAP2; TMEM191C	7	15

The first seven columns represent the chromosome number, inner and outer start and end coordinates of the recurrent CNA region, and the size of the region in base pairs (hg18). The last three columns are the genes encompassed in each CNA region, followed by the sample size (no. of cases) in both Discovery and Validation dataset (Chr: Chromosome; Size 1: Discovery Cluster Size; Size 2: Validation Cluster Size).

**Table 3:** Validated recurrent loss CNA regions with genes

Chr	Inner Start	Inner End	Inner Size	Outer Start	Outer End	Outer Size	Gene Symbol	Size1	Size2
2	97507180	97517476	10296	97507180	97520698	13518	ANKRD36B	5	5
3	62243538	62257523	13985	62242606	62277516	34910	PTPRG	6	6
4	59521	61566	2045	59521	64435	4914	ZNF718; ZNF595	13	6
7	38296343	38297866	1523	38295506	38297939	2433	TRGV11	18	22
8	14388851	14391732	2881	14385622	14391732	6110	SGCZ	23	18
9	5027454	5029342	1888	5027454	5030334	2880	JAK2	7	11
10	89710114	89713882	3768	89708179	89713882	5703	PTENP1; PTEN	10	10
17	21245986	21253816	7830	21227031	21271210	44179	KCNJ12	15	9

The first seven columns represent the chromosome number, inner and outer start and end coordinates of the recurrent CNA region, and the size of the region in base pairs (hg18). The last three columns are the genes encompassed in each CNA region, followed by the sample size in both Discovery and Validation dataset (Chr: Chromosome; Size 1: Discovery Cluster Size; Size 2: Validation Cluster Size).

**Figure 2:**
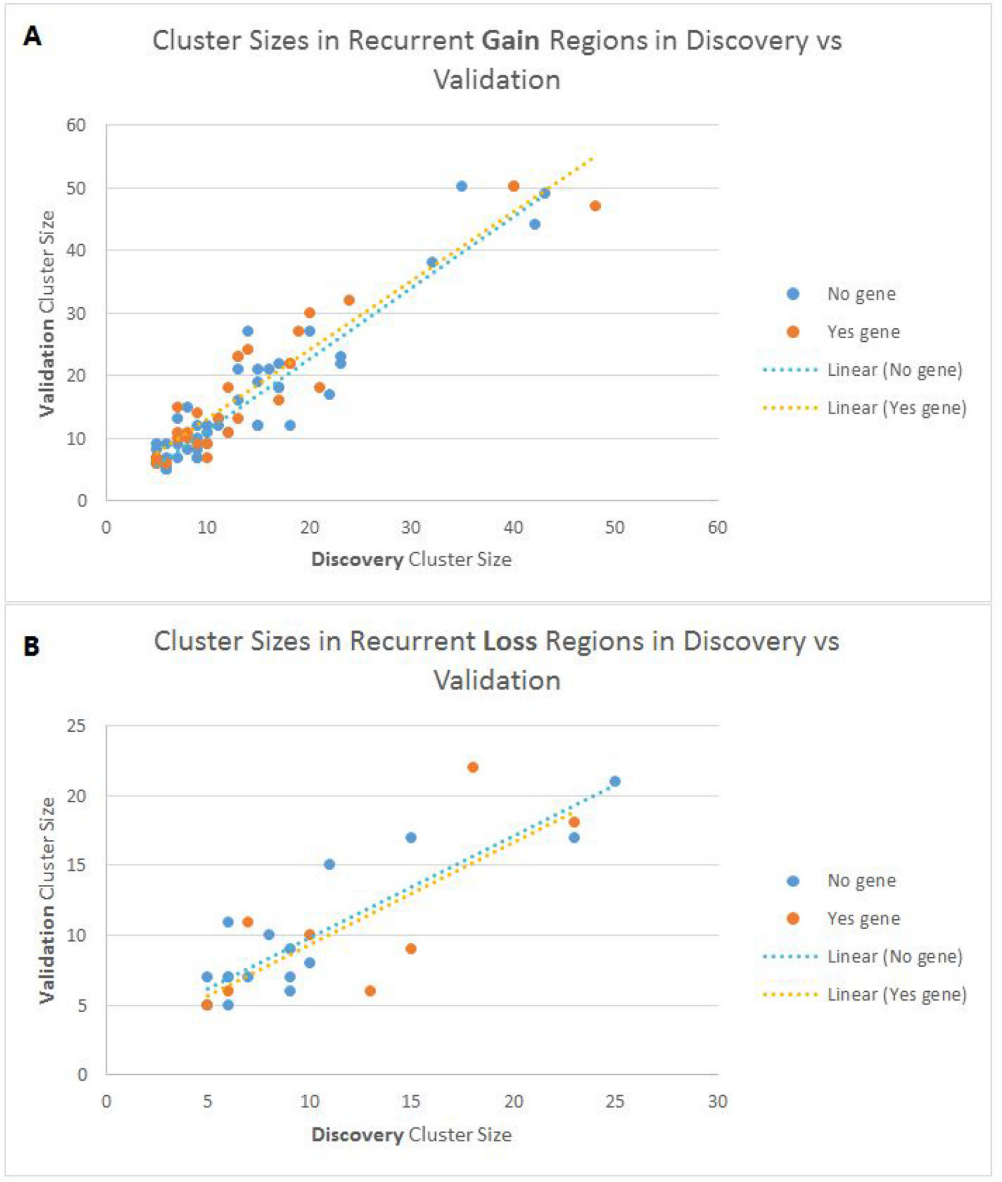
Scatter plot showing the cluster sizes of recurrent CNA regions in the discovery and validation sets (**A**) Gain recurrent young-specific regions and (**B**) Loss recurrent young-specific regions. Each point on the plot represents a young-specific recurrent CNA region. Blue represents regions without genes, and orange represents regions with genes.

Figure 2 shows an overview of how similar the cluster sizes (i.e. number of patients) are in the Discovery set versus the Validation set for all the identified young-specific recurrent CNA regions. It can be seen that for both gain and loss regions, cluster sizes in the Discovery and Validation datasets have a fairly linear relationship. For example, if 30% of the young patients in the Discovery set harbour a CNA region, it is likely that around 30% of patients in the Validation set will harbour that region as well.

### Annotation of the identified recurrent CNA regions

**Figure 3:**
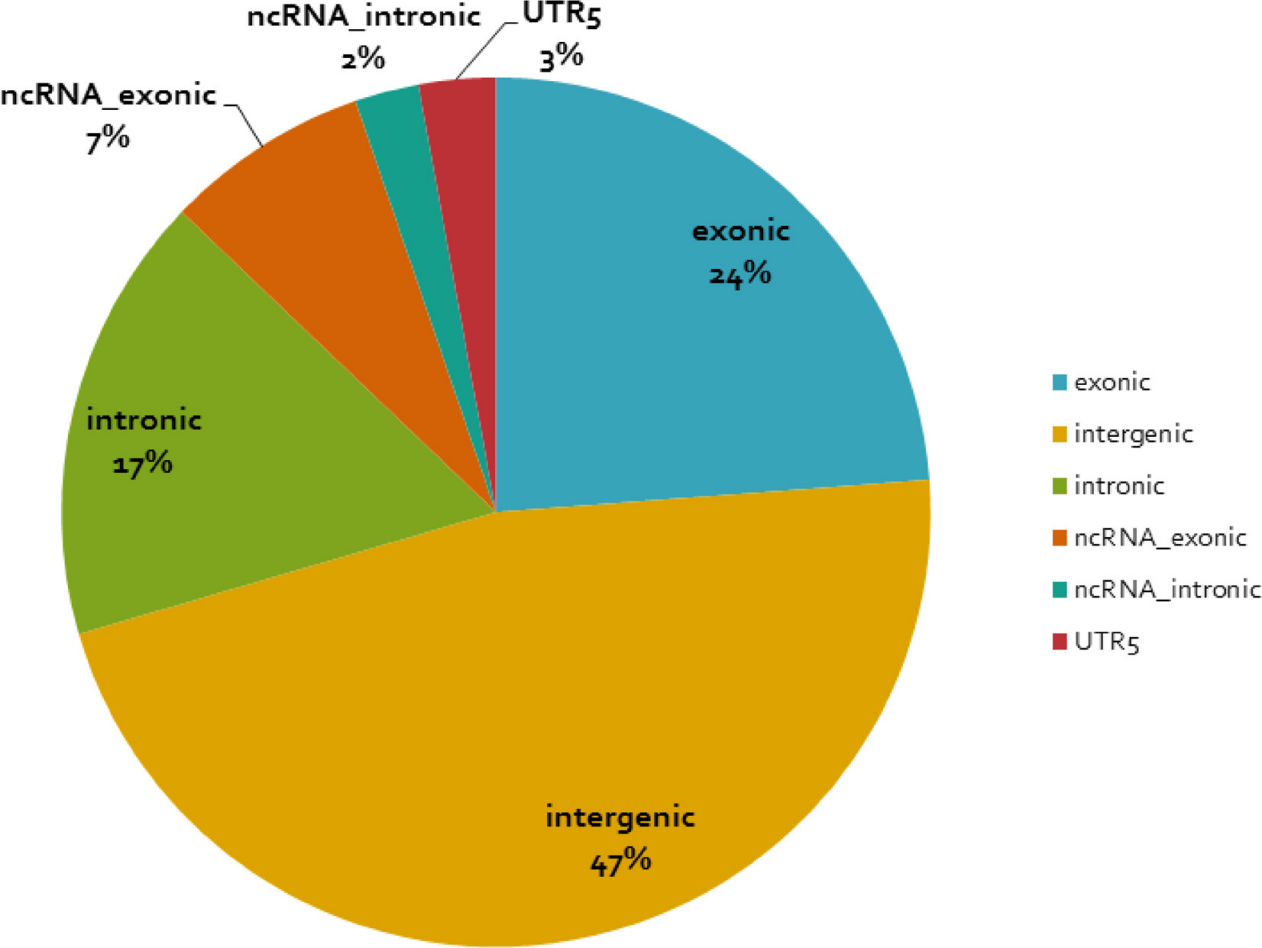
Distribution of the identified young-specific recurrent CNA regions with respect to the genome location The functional annotation of the young-specific recurrent CNA regions is based on software ANNOVAR.

We performed region-based variation annotation on the identified young-specific recurrent CNA regions (see Tables 2 and 3 and Supplementary Tables 1 and 2) with refGene using the software ANNOVAR (Annotate Variation). The complete annotation information of the recurrent CNA regions is shown in Supplementary Table 3. Figure 3 shows the genome location distribution of our recurrent CNAs with respect to the encompassed regions. The majority of the CNAs are in non-coding regions (76%) and 24% in coding regions.

**Figure 4:**
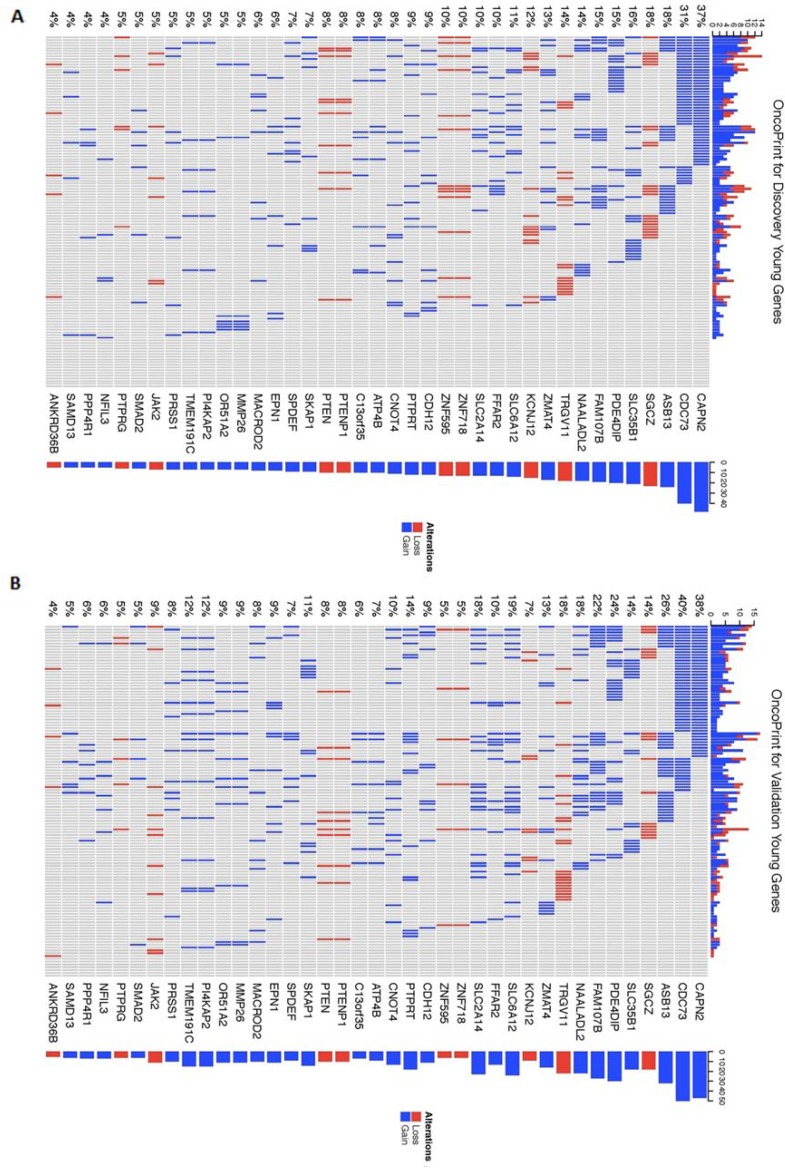
Heatmap of mutation distribution for genes identified in the recurrent young-specific CNA gain and loss regions (**A**) Results from the Discovery dataset and (**B**) Results from the Validation dataset. Rows are sorted based on the frequency of the alterations in all young-specific samples and columns are sorted to visualize the mutual exclusivity across genes. Barplots at both sides of the heatmap show numbers of different alterations for each sample and for each gene. Red represents CNA loss mutations and blue represents CNA gain mutations.

In order to better visualize the mutation distribution of the 39 genes encompassed in the recurrent CNAs identified in the coding regions in both the Discovery and Validation young women group, an R package called the ComplexHeatmap was applied (Figure 4). From the heatmap, it can be observed that CNA gain regions encompassing genes *CAPN2, CDC73* and *ASB13* are the top 3 most frequent in both Discovery and Validation dataset (young women age group), while gene *SGCZ* ranks top for recurrent CNA loss regions in the two datasets.

### Expression quantitative trait locus analysis

**Figure 5:**
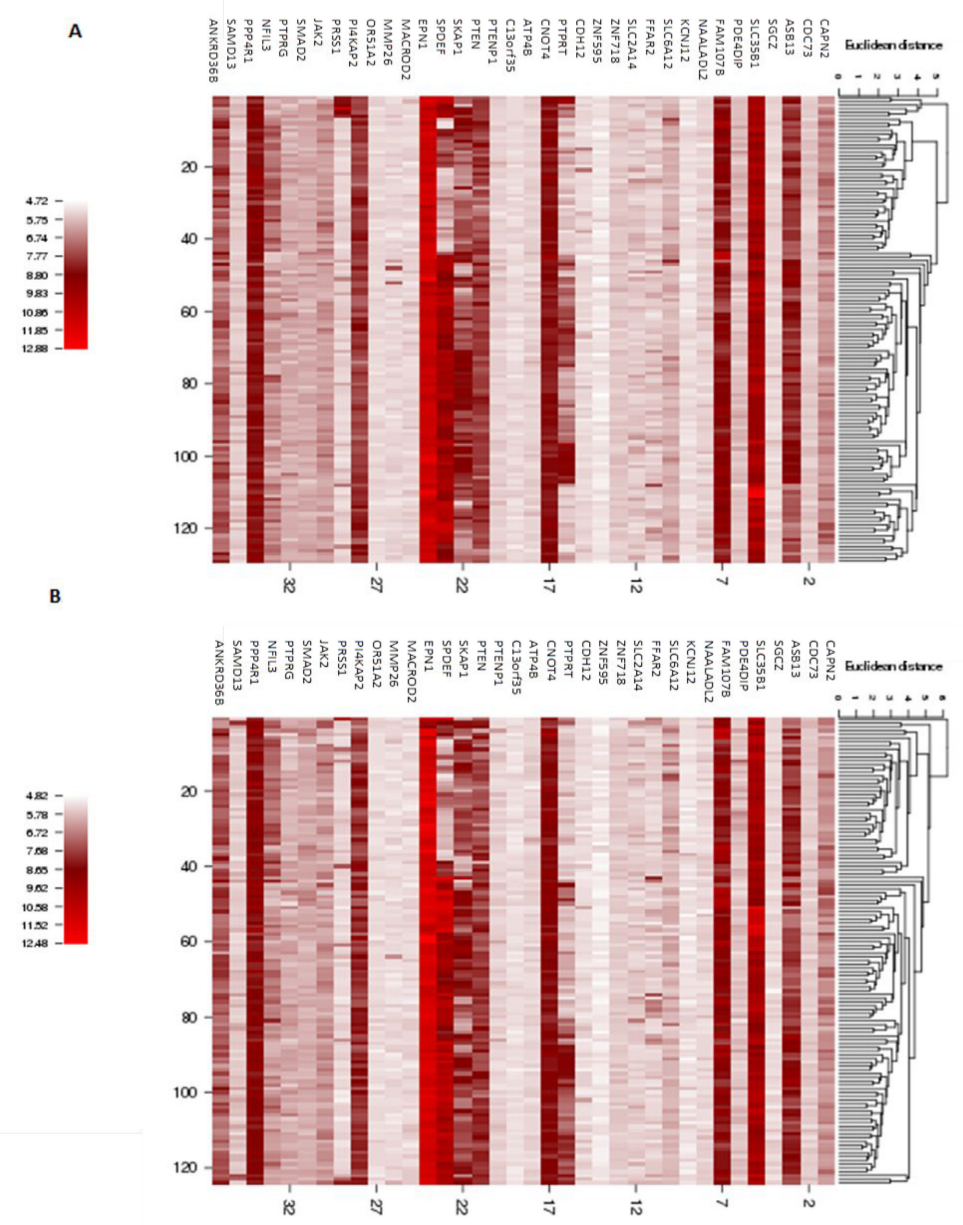
Heatmap of gene expression for the genes identified in the recurrent young-specific CNA regions in young breast cancer patients (**A**) Results from the Discovery dataset (**B**) Results from the Validation dataset. Rows represent the gene expression levels for the genes identified in the recurrent young-specific CNA gain and loss regions (same order as in Figure 4 for comparison). Columns represent the young-specific samples in Discovery and Validation datasets. The higher the intensity of the red colour, the higher the gene expression level.

An overview of the expression levels for each of the identified young-specific genes across all the young patients samples in the Discovery (Figure 5A) and Validation datasets (Figure 5B) is provided as gene expression heatmaps. Further interrogation using logistic regression was performed to evaluate the statistical association between gene expression and CNA mutation status (Table 4). In total, 16 gain regions and 1 loss region show significant associations with their gene expression changes. However, the directionality of the association is ambiguous. Fourteen out of the 16 gain regions correlated with high gene expression while the other 2 gain regions (encompassing *MMP26* and *SPDEF*) were associated with low gene expression. For example, mutated gain CNA status in *ASB13* seems to lead to higher gene expression. On the other hand, the loss region encompassing *PTEN* was found to be associated with having high gene expression level.

**Table 4:** Logistic regression analysis between CNA mutation status and gene expression in combined dataset

Gene Symbol	Copy Number State	*P*-value	Odds Ratio^†^	Discovery Sample Size	Validation Sample Size
ASB13	Gain	0.049	1.83 (1.00–3.34)	24	32
ATP4B	Gain	0.021	3.51 (1.21–10.17)	10	9
CAPN2	Gain	0.00002	3.21 (1.89–5.45)	48	47
CDH12	Gain	0.026	2.87 (1.14–7.24)	12	11
CNOT4	Gain	0.0004	7.45 (2.46–22.49)	11	13
EPN1	Gain	0.004	9.57 (2.15–42.56)	8	11
PDE4DIP	Gain	0.002	2.73 (1.45–5.15)	20	30
PI4KAP2	Gain	0.031	2.81 (1.10–7.14)	7	15
PPP4R1	Gain	0.016	6.7 (1.44–31.23)	5	7
SLC35B1	Gain	0.001	19.19 (6.54–56.27)	21	18
SMAD2	Gain	0.022	6.06 (1.30–28.22)	6	6
SPDEF	Gain	0.057	0.39 (0.15–1.03)	9	9
FAM107B	Gain	0.083	1.79 (0.93-3.46)	19	27
MACROD2	Gain	0.085	2.73 (0.87-8.55 )	8	10
MMP26	Gain	0.098	0.43 (0.15-1.17)	7	11
NFIL3	Gain	0.068	3.46 (0.91-13.08)	5	7
PTEN	Loss	0.002	29.12 (3.83–221.25)	10	10

^†^Odds ratio is followed by its corresponding 95% confidence interval in brackets.

### Survival analysis

We further evaluate whether the expression levels of these genes are associated with disease-specific survival (DSS) (Table 5). The expression levels of eight out of the 39 young-specific genes are significantly associated with survival outcome. A higher gene expression of genes *CAPN2*, *NFIL3* and *SLC35B1* was associated with a moderately worse survival outcome.

**Table 5:** Cox proportional hazard analysis of disease (breast cancer) specific survival of gene expression in combined dataset (discovery and validation)

Genes	CNA types	*P*-value	Hazard ratio^†^	Discovery Sample Size	Validation Sample Size	Chr	InnerStart	InnerEnd
ASB13	Gain	0.0001	0.54 (0.39–0.73)	24	32	10	5737990	5742226
CAPN2	Gain	0.091	1.54 (0.93–2.54)	48	47	1	222004315	222004925
NFIL3	Gain	0.009	1.58 (1.13–2.23)	5	7	9	93166194	93261927
PDE4DIP	Gain	0.027	0.37 (0.16–0.89)	20	30	1	143607802	143609034
PTPRT	Gain	0.0002	0.68 (0.55–0.83)	12	18	20	41215727	41219453
SKAP1	Gain	0.0002	0.66 (0.53–0.82)	9	14	17	43751830	43753351
SLC35B1	Gain	0.00005	1.88 (1.39–2.55)	21	18	17	45136676	45139395
JAK2	Loss	0.007	0.43 (0.24–0.79)	7	11	9	5027454	5029342

^†^Hazard ratio is followed by its corresponding 95% confidence interval in brackets.

**Figure 6:**
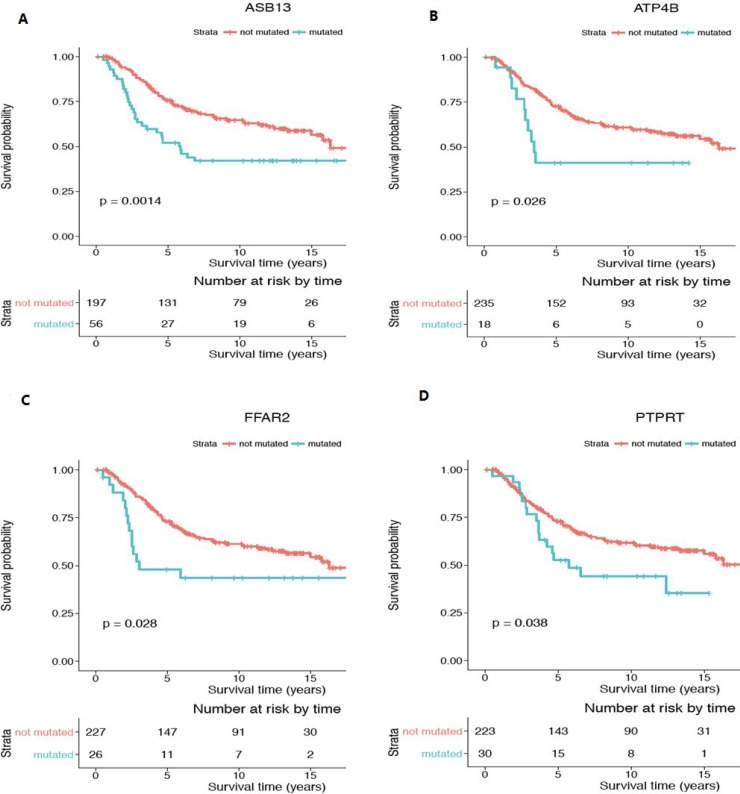
Kaplan-Meier survival analysis for genes with significant CNA gain mutations in the young women group Genes showing with statistical significance (*p* < 0.05) are (**A**) ASB13, (**B**) ATP4B, (**C**) FFAR2 and (**D**) PTPRT. Survival curve in red represents patients without CNA mutation in the corresponding gene (CN = 2) while the curve in blue represents patients with CNA gain mutations in the corresponding gene (CN > 2). Y-axis is the cumulative survival probability and X-axis is the survival time in years.

**Figure 7:**
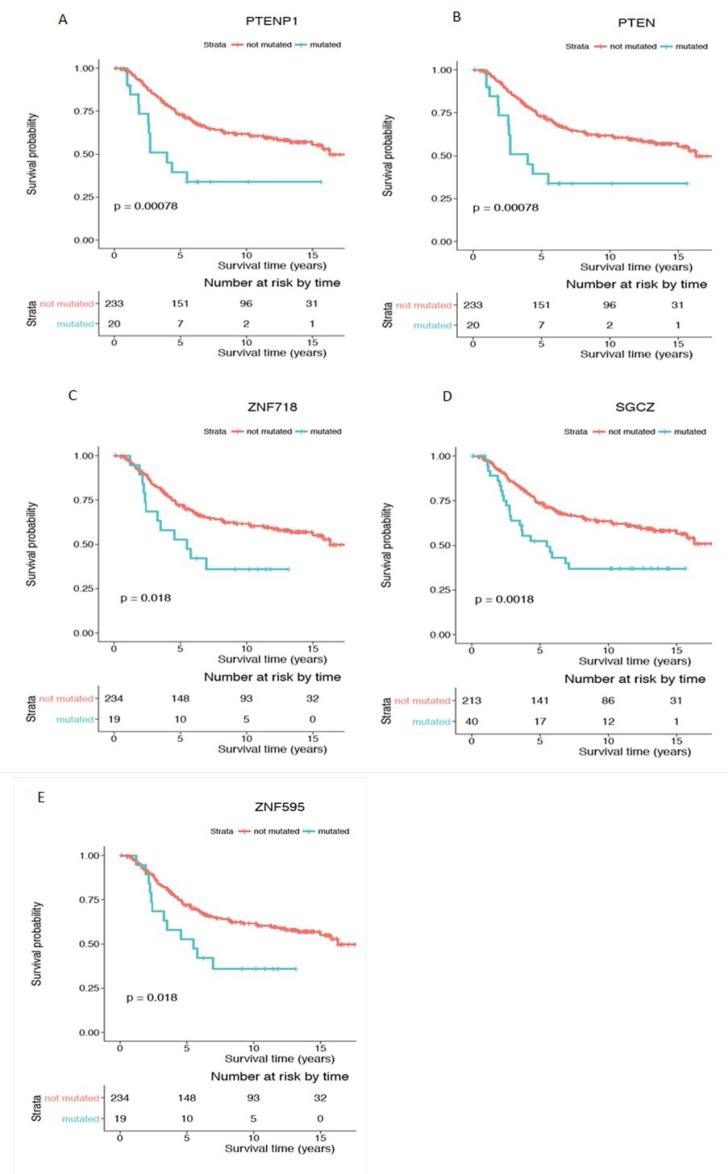
Kaplan-Meier survival analysis for genes with significant CNA loss mutations in the young women group Genes showing statistical significance (*p* < 0.05) are (**A**) PTENP1, (**B**) PTEN, (**C**) ZNF718, (**D**) SGCZ and (**E**) ZNF595. Survival curve in red represents patients without CNA mutation in the corresponding gene (CN = 2) while the curve in blue represents patients with CNA loss mutations in the corresponding gene (CN < 2). Y-axis is the cumulative survival probability and X-axis is the survival time in years.

Of particular interest, the mutation status of two genes, *ASB13* (Figure 6A) and *SGCZ* (Figure 7D), was also significant in the Kaplan Meier survival analysis, which allows estimation of a survival curve over time. Patients with a mutated status in both of these genes resulted in a worse survival outcome when compared to patients without the gene mutations. Other genes found to be significant in the survival analysis include *ATP4B* (Figure 6B), *FFAR2* (Figure 6C) and *PTPRT* (Figure 6D), all encompassed within CNA gain regions. *PTENP1* (Figure 7A), *PTEN* (Figure 7B), *ZNF718* (Figure 7C) and *ZNF595* (Figure 7E), all encompassed in CNA loss regions.

### Cancer-relevant candidate genes

### PTEN (Phosphatase and tensin homolog)

Results from our study show that the median survival time (i.e. half of the patients are expected to be alive) for young patients with a copy number loss in the *PTEN* gene region is ~4 years as opposed to ~15 years for those without. *PTEN* (cytoband 10q23.31) has been identified as a tumour suppressor which inhibits the PI3K/Akt/mTOR signalling pathways [23]. It has been shown to be one of the most frequently mutated genes in all cancer types, including that of breast, ovary, prostate, glioblastoma and lymphoma. Previous studies have observed that 40% of invasive breast cancers have a loss of *PTEN* heterozygosity, and that the loss of one gene copy is sufficient to disrupt cell signalling and cell growth control. It has also been suggested that carriers of the *PTEN* mutation are at higher risk of developing breast cancers at a younger age [24].

### SGCZ (sarcoglycan zeta)

Our study shows that ~16% of all young patients present a CNA loss mutation encompassing *SGCZ*, with a significantly shorter median survival time for young patients with this mutation of ~6 years in contrast to ~15 years for those without. *SGCZ* (8p22) encodes a protein that is part of the sarcoglycan complex, which plays a role in connecting the inner cytoskeleton to the extracellular matrix, possibly maintaining membrane stability [25]. Although the exact function of *SGCZ* in cancer is not well understood, loss of the chr8p region has been associated with several factors involved in cancer development and progression, such as the tumour having an aggressive histology, increased cell proliferation, and large size as well as the patients having increased early recurrence rate and mortality, and overall poor survival in young women. This region also contains the gene *DLC1* (deleted in liver cancer 1), which has been suggested to act as a tumour suppressor [26]. DLC1 encodes a GAP protein that inhibits the activation of Rho-GTPases, which are often associated with a loss of cell adhesion. *DLC1* expression has been reported to be frequently lost in tumour cells, leading to a constitutive activation of the Rho-GTPases.

### CAPN2 (calpain 2)

CAPN2 (cytoband 1q41) was the most frequent CNA gain mutation in our study, with ~37-38% of all young patients harbouring a CAPN2 gain mutation. Calpains are calcium-activated intracellular proteases that have the ability to cleave cytoskeletal proteins, possibly playing a role in regulating cell invasion and migration [27]. A knockdown study of CAPN2 in breast tumour cells resulted in reduced cell migration, proliferation, as well as reduced Akt activation, increased FoxO nuclear localization and p27 expression [27]. It was suggested that CAPN2 promotes cell proliferation through the Akt-FoxO-p27 signalling pathway.

### NAALADL2 (N-acetyl-L-aspartyl-L-glutamate peptidase-like 2)

Our study shows that ~16% of all young patients present a CNA gain mutation encompassing NAALADL2. NAALADL2 is a member of the NAALADase protein family which act as matrix metalloproteases and have the ability to alter the tumour environment. Microarray studies have shown that NAALADL2 is often overexpressed in prostate and colon cancers and stimulates a migratory and metastatic phenotype. A proposed mechanism is that since NAALADL2 has been found to be basal-localized, it may enhance interaction of tumour cells with the extracellular matrix surrounding the tumour and provide a mechanism for the tumour cells to escape [28]. Subsequent survival analysis shows that patients with NAALADL2 overexpression have a 45% chance of surviving up to 5 years as opposed to 93% for patients with low NAALADL2 expression. It remained prognostic for recurrence rate even after correction for clinical variables such as tumour stage and grade. Expression array analyses also associated its overexpression to changes in the epithelial-to-mesenchymal transition (EMT) and cell adhesion pathways.

### Pathway enrichment analysis

A pathway enrichment analysis using the ANNOVAR gene list (174 genes) via the Enrichr REACTOME database reveals a significant overrepresentation of phospholipid signaling (*MTMR14*,*PTEN*,*PIP4K2A*) and adherens junction (*CDH12*, *CDH18*, *CDH7*) pathways (*p* < 0.05) in the identified young-specific recurrent CNA regions with genes. Both enriched pathways are highly relevant to cancer development and progression.

### Phospholipid signaling

Aside from playing an important role in structural components, lipids also have a role in signalling processes [29, 30]. These lipid molecules aggregate to form lipid rafts as highly specific platforms for cell signalling, carrying signals from activated growth factor receptors to the intracellular machinery [31]. These receptors recruit signalling effectors that induce cell proliferation and reduce cell death, dysregulation of which contributes to cancer development and progression. The phosphatidylinositol (3,4,5)-trisphosphate molecule, also known as PIP3, is generated by PI3K and leads to activation of downstream signaling components. A well-known consequence is recruitment and activation of protein kinase Akt, which can phosphorylate a variety of substrates, which in turn activate cell growth, apoptosis and cell cycle processes. PIP3 is a substrate for phosphatase and tensin homologue (PTEN), which is required for dephosphorylation of PIP3 into PIP2, essential for inhibition of the AKT pathway. Dysregulation of these pathways is frequent in many cancer types.

### Cell adhesion

Cellular adhesion plays a major role in maintaining the integrity of normal cell-cell connections, and disruption in this pathway has been strongly associated with metastasis in cancers. Adherens junctions, which are sites of intracellular signalling and anchoring, provide strong bonds between adjacent cell membranes. The molecular processes governing cell-cell adhesion are very finely controlled, since they inhibit epithelial-mesenchymal transition (EMT) that is normally present during embryogenesis and tissue repair. Characteristics of EMT include a loss in intercellular adhesion and enhancement of cell migration, leading to a more motile phenotype [32]. Notably, the adherens junctions are lost during the process of EMT, which increases the risk of cancer progression such as metastasis. In normal tissues, epithelial cells are tightly bound to one another. However, in advanced cancer, many epithelial tumour cells show loss of cell-cell adhesion and increased tissue invasion. Tumours featuring local spreading and invasion are suggested to have a more aggressive phenotype and be associated with a higher mortality rate of the patient. This phenomenon has been widely seen in various cancer types, including breast, colon, prostate, ovarian and other types of cancer [33].

## CONCLUSIONS

Applying the graph-based algorithm to the Molecular Taxonomy of Breast Cancer International Consortium (METABRIC) breast cancer dataset, we have identified and validated 81 recurrent CNA gain regions and 25 validated recurrent CNA loss regions specific to young-Women’s breast cancers. As well, we have located the corresponding candidate protein encoding genes that are encompassed in these regions. The graph-based algorithm guarantees that the identified CNA regions are the most frequent and that the minimal regions have been delineated.

Identification of molecular alterations associated with disease outcome may improve risk assessment and treatments for aggressive breast cancer, especially for young women. It can give new insights into the role of CNAs in cancer predisposition, development and progression as well as contribute to a more accurate and complete human cancer genome sequence reference. We hope that the results of this study will in the future, facilitate the development of screening methods for breast cancer biomarker discovery, especially in young women, as more prospective samples become available.

Since CNAs are fairly large in size, in the future it would be interesting to characterize further the non-coding CNA regions we have identified and their role in regulating gene expression levels either in cis or trans.

## MATERIALS AND METHODS

### Data source

All breast cancer data are retrieved from the Molecular Taxonomy of Breast Cancer International Consortium (METABRIC) [22], which is a novel dataset consisting of comprehensive clinical features such as breast cancer-specific survival data, PAM50 subtypes, ER/PR/HER2 status, tumour grade and tumour sizes. Each case has corresponding whole gene expression profiles (Illumina HT-12 v3 platform), SNPs and somatic DNA copy-number profiling data (Affymetrix Human SNP 6.0 platform). Treatments for the patients are homogeneous among each clinically relevant group: almost all ER-positive/LN-negative patients did not receive chemotherapy, while ER-negative/LN-positive patients did receive chemotherapy. Furthermore, the METABRIC cohort consists of cohorts prior to the usage of Herceptin/trastazumab in standard clinical care. Therefore, the outcome of HER2 positive patients reflects the poor prognosis in such patients before the introduction of this targeted therapy [22].

All samples are derived from ~2,000 clinically annotated primary fresh-frozen breast cancer specimens from tumour banks in the UK and Canada (a discovery set of 997 primary tumours and a validation set of 995 tumours were divided by METABRIC). All genomic and clinically annotated data are available at the European Genome-Phenome Archive (http://www.ebi.ac.uk/ega/), under accession number EGAS00000000083 [22]. The individual CNA calls of the ~2,000 individual samples are pre-existing from the METABRIC study and downloaded from EGAS00000000083 [22]. Circular binary segmentation (CBS) method is used for making individual CNA calls. CBS is a segmentation-based method that scans for change points in an ordered sequence of copy number values to delineate segments with different distribution of the values (measured by having different means). In other words, it will recursively divide up the chromosome until segments that have probe distribution different than neighbours have been identified [41].

### Representing CNAs as an interval graph

**Figure 8:**
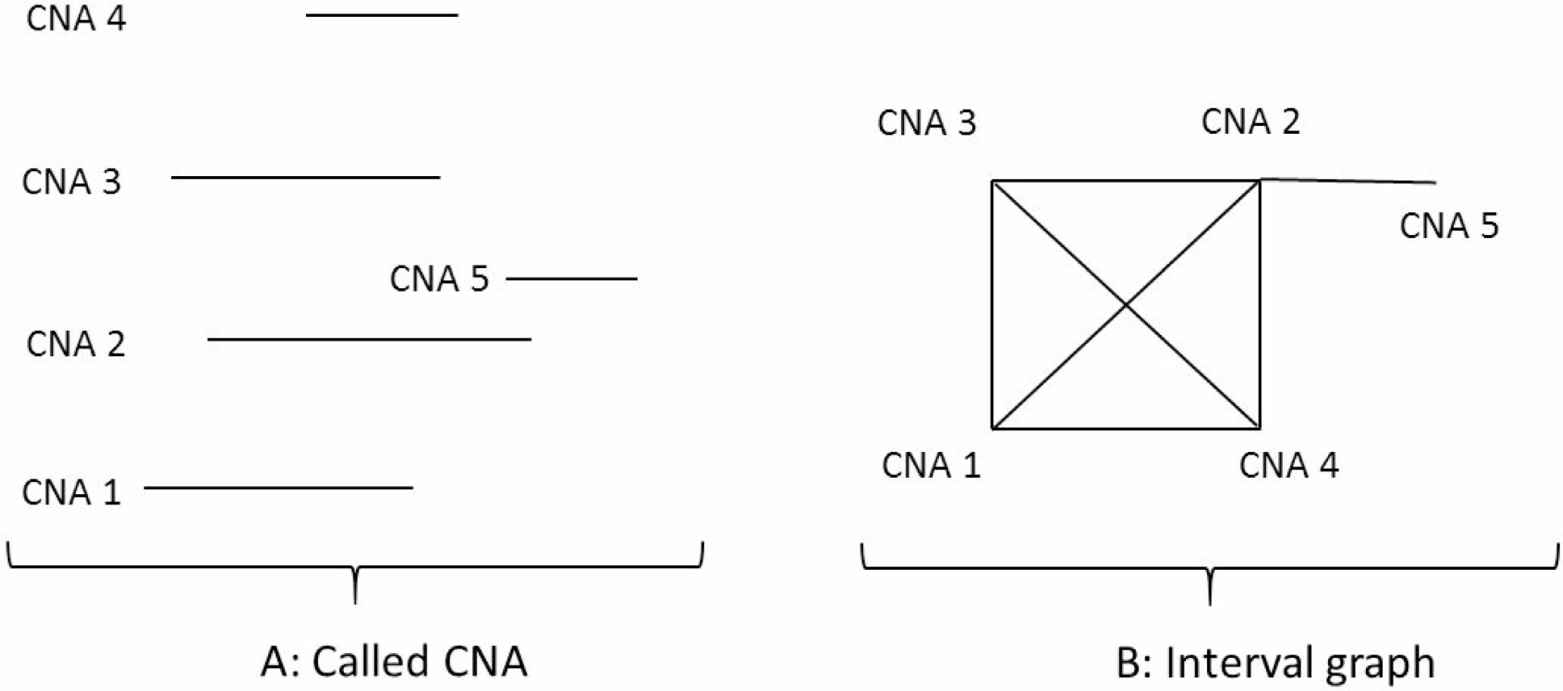
Representing CNAs as an interval graph (**A**) CNA 1, CNA 2, CNA 3, CNA 4, CNA 5 are individual patient level CNAs on a specific chromosome. Each of the CNAs has chromosome start and end positions. (**B**) This is an interval graph where CNA 1, CNA 2, CNA 3, CNA 4, CNA 5 are the individual patient level CNAs in (A). The edge between each of two vertices in the graph represents the two individual patient level CNAs sharing a piece of common regions on the chromosome.

Figure 8A shows examples of five individual patient level CNA segments (CNA 1, CNA 2, CNA 3, CNA 4, CNA 5) on the same chromosome. Each of the five CNAs contains chromosomal-specific start (left) and end (right) positions. To identify the common regions of individual patient level CNAs on the same chromosome, the intersection among the individual patient level CNAs can be represented as an interval graph, treating each called individual patient level CNA as a vertex of the graph and connecting two vertices only if the corresponding intervals have an intersecting region. Thus, the constructed interval graph *G*(*V, E*) is comprised of a set of vertices *V*, where each vertex (*v* ϵ *V*) corresponds to a specific interval of the individual patient level CNA and each edge ({*u*, *v*} ϵ *E*) connects two intersecting intervals *u* and *v*. In Figure 8B, an example of the interval graph is shown where CNA 1 through CNA 4 are the intervals (nodes of the graph or individual patient level CNAs) and an edge connects two nodes (individual patient level CNAs) if the intervals overlap.

To find maximal cliques in an interval graph constructed from individual patient level CNAs, we applied Gentlemen and Vandal’s algorithm [34]. The main idea of the algorithm is to sort the vertices based on their chromosomal end positions. The ordering is important because it allows the algorithm to discard vertices in each iteration without losing the triangulation property. The input of the algorithm is the individual patient level CNAs on a specific chromosome, which includes two parameters for each CNA segment: start and end positions (base pair).

Each of the identified maximal cliques is a recurrent CNA, which is common in multiple patients. The shared region of the recurrent CNA across multiple patients is the minimal common region (MCR) of the CNA, which has the potential to harbour cancer causing/associated genes. In practice, the size of the maximal cliques should be at least 2 and the size of the MCRs should be at least 1kb. It should be noted that we need to analyse CNA gains and losses separately. More details of the algorithm and its applications can be found in [35].

### Survival analysis

Disease (breast cancer) specific survival analysis was performed for both the mutation status (CNA gain, CNA loss) by the product-limit method or The Kaplan-Meier method and the expression level of the corresponding genes that are encompassed in the validated recurrent CNA regions using the Cox proportional hazard model [36]. All analyses were performed using Survival R package (https://cran.r-project.org/web/packages/survival/index.html).

### eQTL analysis

An expression quantitative trait locus (eQTL) is a locus that explains a portion of the genetic variance of a gene expression phenotype. An eQTL analysis tests for direct associations between markers of genetic variation with gene expression levels; that is, to evaluate the association between gene expression and CNA mutation status. Logistic regression is used to estimate the probability p associated with a dichotomous response for various values of an explanatory variable. In this case, the response (dependent) variable is gene expression (binarized-by-mean) and the predictor (independent) variable is CNA status.

### Functional analysis

Functional analysis such as enrichment and annotations have been carried out using software (Enrichr and ANNOVAR) to determine whether the identified CNA regions with protein coding genes are enriched in any interesting pathways or functions. Enrichr software [37] contains a diverse and up-to-date collection of over 100 gene-set libraries available for analysis and download. It is used to perform pathway enrichment analysis on the identified young-specific genes to identify which pathways are over-represented in the gene-set. ANNOVAR [38] is a perl command line program for genome annotation. This region-based annotation is used to identify affected genomic regions that lie outside of the protein-coding regions.

### Biological visualization

In order to aid in clearer visualization of and assist interpretation of the results, software programs Oncoprint [39] and CIMminer [40] were used to generate heatmap visualizations for the identified candidate regions. Oncoprint is included in the R package ComplexHeatMap, and it is a way to visualize multiple genomic alteration events in the format of a heatmap. This is used to visualize the frequencies of CNA mutation for each of the young-specific regions with genes in Discovery and Validation datasets. CIMminer generates color-coded Clustered Image Maps (CIMs) to portray “high-dimensional” data sets such as gene expression profiles. It is used to visualize the relative expression levels in terms of colour intensity for each of the identified young-specific genes.

## SUPPLEMENTARY MATERIALS AND TABLES







## Abbreviations

CBS: Circular binary segmentation
ANNOVAR: Annotate Variation
BC: Breast cancer
CNA: Copy number alteration
CIM: Clustered Image Map
eQTL: Expression quantitative trait locus
EMT: Epithelial-mesenchymal transition
ER: Estrogen
GWAS: Genome-wide association studies
METABRIC: Molecular Taxonomy of Breast Cancer International Consortium
PR: Progesterone receptor
