# Supplementary material for: Recurrent copy number alterations in young women with breast cancer
Source: Oncotarget. 2018 Jan 29;9(14):11541–58. doi: 10.18632/oncotarget.24336 (PMC5837756; doi:10.18632/oncotarget.24336)
Supplement: Supplementary file 1 [file oncotarget-09-11541-s001.pdf]

Recurrent copy number alterations in young women with breast cancer

SUPPLEMENTARY MATERIALS

Supplementary Table 1: Validated recurrent gain CNA regions without genes. The first seven columns represent the chromosome number, inner and outer start and end coordinates of the recurrent CNA region, and the size of the region in base pairs (hg18). The last two columns are the sample sizes in both Discovery and Validation dataset, respectively. See Supplementary_Table_1

Supplementary Table 2: Validated recurrent loss CNA regions without genes

Chromosome	InnerStart	InnerEnd	Inner.CNV. Size	OuterStart	OuterEnd	Outer. CNV.Size	Discovery. Cluster. Size	Validation. Cluster.Size
1	16885044	16885329	285	16884038	16889666	5628	11	15
3	26589981	26589990	9	26588205	26591799	3594	7	7
3	64855417	64863915	8498	64731448	64923169	191721	6	5
4	104455633	104467384	11751	104414905	104467384	52479	5	5
4	157100338	157107691	7353	157100332	157107969	7637	5	7
5	60888563	61017766	129203	60887533	61460364	572831	8	10
8	3552110	3554053	1943	3551271	3564930	13659	25	21
8	5633227	5636521	3294	5625845	5636589	10744	23	17
9	1490330	1490755	425	1456070	1504392	48322	6	7
9	1500170	1503092	2922	1490330	1504392	14062	6	7
13	22518343	22523101	4758	22512167	22523373	11206	10	8
13	56656317	56661149	4832	56656291	56661535	5244	15	17
18	36514797	36519362	4565	36514489	36519388	4899	9	6
18	61883719	61883744	25	61878473	61885427	6954	5	5
22	20816553	20820788	4235	20816118	20833270	17152	9	9
15	22859182	22873331	14149	22846930	22875304	28374	6	11
17	33672360	33694008	21648	33664567	33752586	88019	9	7

The first seven columns represent the chromosome number, inner and outer start and end coordinates of the recurrent CNA region, and the size of the region in base pairs (hg18). The last two columns are the sample sizes in both Discovery and Validation dataset, respectively.

Supplementary Table 3: Functional annotation of the validated recurrent CNA regions. The identified recurrent CNA gain and loss regions are annotated for both inner regions (intersection region of all individual CNAs in a cluster) and outer regions (union region of all individual CNAs in a cluster) using software ANNOVAR. See Supplementary_Table_3
